# Fibroblast Growth Factor 23 Expression Is Increased in Multiple Organs in Mice With Folic Acid-Induced Acute Kidney Injury

**DOI:** 10.3389/fphys.2018.01494

**Published:** 2018-10-23

**Authors:** Daniela Egli-Spichtig, Martin Y. H. Zhang, Farzana Perwad

**Affiliations:** Department of Pediatrics, Division of Nephrology, University of California, San Francisco, San Francisco, CA, United States

**Keywords:** fibroblast growth factor 23, acute kidney injury, 1, 25(OH)_2_D, inflammation, vitamin D receptor, tumor necrosis factor

## Abstract

Fibroblast growth factor 23 (FGF23) regulates phosphate homeostasis and vitamin D metabolism. In patients with acute kidney injury (AKI), FGF23 levels rise rapidly after onset of AKI and are associated with AKI progression and increased mortality. In mouse models of AKI, excessive rise in FGF23 levels is accompanied by a moderate increase in FGF23 expression in bone. We examined the folic acid-induced AKI (FA-AKI) mouse model to determine whether other organs contribute to the increase in plasma FGF23 and assessed the vitamin D axis as a possible trigger for increased *Fgf23* gene expression. Twenty-four hours after initiation of FA-AKI, plasma intact FGF23 and 1,25(OH)_2_D were increased and kidney function declined. FA-treated mice developed renal inflammation as shown by increased *Tnf* and *Tgfb* mRNA expression. *Fgf23* mRNA expression was 5- to 15-fold upregulated in thymus, spleen and heart of FA-treated mice, respectively, but only 2-fold in bone. Ectopic renal *Fgf23* mRNA expression was also detected in FA-AKI mice. Plasma FGF23 and *Fgf23* mRNA expression in thymus, spleen, heart, and bone strongly correlated with renal *Tnf* mRNA expression. Furthermore, *Vdr* mRNA expression was upregulated in spleen, thymus and heart and strongly correlated with *Fgf23* mRNA expression in the same organ. In conclusion, the rapid rise in plasma FGF23 in FA-AKI mice is accompanied by increased *Fgf23* mRNA expression in multiple organs and increased *Vdr* expression in extra osseous tissues together with increased plasma 1,25(OH)_2_D and inflammation may trigger the rise in FGF23 in FA-AKI.

## Introduction

Fibroblast growth factor 23 (FGF23) is a potent regulator of phosphate homeostasis and vitamin D metabolism (Shimada et al., 2004). FGF23 binds to its co-receptor αKlotho and FGFR receptors 1c, 3 and 4 to activate mitogen activated protein kinase (MAPK) signaling pathway (Kurosu et al., 2006; Ranch et al., 2011; Chen et al., 2018). In the kidney, FGF23 inhibits phosphate reabsorption and suppresses 1,25-dihydroxyvitamin D (1,25(OH)_2_D) production by downregulation of *Cyp27b1* and upregulation of *Cyp24a1* gene expression (Shimada et al., 2004; Perwad et al., 2007). *Cyp27b1* and *Cyp24a1* encode the enzymes 1α-hydroxylase and 24-hydroxylase, respectively, which are responsible for 1,25(OH)_2_D synthesis (Takeyama et al., 1997) and degradation (Ohyama et al., 1991). FGF23 is mainly expressed in bone but low levels of expression are detected in thymus, spleen and heart (Fon Tacer et al., 2010).

In patients with acute kidney injury (AKI), plasma and urinary C-term FGF23 (cFGF23) levels rise rapidly after onset of AKI and are independently associated with AKI progression and adverse outcomes (Leaf et al., 2013, 2016, 2017). Increase in cFGF23 levels is independent of changes in plasma PTH, phosphate and vitamin D metabolites (Leaf et al., 2016). In pediatric and adult patients with AKI following cardiac surgery, there is a transient increase in plasma intact FGF23 (iFGF23) levels which subsequently normalize (Hanudel et al., 2016; Leaf et al., 2016). Preoperative plasma cFGF23 levels in pediatric and adult patients undergoing cardiac surgery are associated with increased risk of AKI and post-operative mortality (Speer et al., 2015; Hanudel et al., 2016). Although, pre- and post-operative cFGF23 levels serve as prognostic tools to predict post-operative AKI severity and complications (Speer et al., 2015; Leaf et al., 2016), the mechanisms by which FGF23 levels increase in AKI are poorly understood.

In the folic acid-induced AKI (FA-AKI) mouse model, plasma FGF23 levels rise rapidly after onset of AKI and are partially independent of phosphate and 1,25(OH)_2_D suggesting that there are other contributing mechanisms (Christov et al., 2013). FGF23 production is elevated in bone of FA-AKI mice (Christov et al., 2013) and systemic FGF receptor blockade reduces bone *Fgf23* mRNA expression and normalizes FGF23 levels (Hassan et al., 2016). Considering that FGF23 production in bone is only modestly increased in FA-AKI, other organs may contribute to the massive increase in FGF23 levels (Christov et al., 2013; Hassan et al., 2016). In hemorrhagic shock and sepsis-induced AKI rat model, *Fgf23* gene expression is increased in bone marrow and is erythropoietin (EPO)-dependent (Toro et al., 2018).

In this study, we determined whether other organs contribute to the increase in plasma FGF23 levels in the FA-AKI mouse model and assessed the vitamin D axis as possible mechanism for stimulation of FGF23 production in multiple organs.

## Methods

### Animals

Eight-week-old male and female C57Bl/6J mice from in house breeding were used for all experiments. Animals were allowed *ad libitum* access to water and food (PicoLab Mouse Diet 20 5058). AKI was induced by intraperitoneal injection of folic acid (250 mg/kg; F7876, Sigma-Aldrich) or vehicle (0.15 M NaHCO_3_) as previously described (Christov et al., 2013). After 24 h, mice were anesthetized with Ketamine/Xylazine before blood and organs were collected. All animal studies were performed according to approved protocols (Institutional animal care and use committee, San Francisco).

### Plasma Analysis

Blood was drawn from the heart and collected in BD Microtainer^TM^ Tubes containing Lithium Heparin (Becton, Dickinson and Company) for plasma separation. Plasma was aliquoted, rapidly frozen and stored at −80°C. Plasma phosphate, creatinine and BUN were measured with Phosphorus Liqui-UV^®^ test, Creatinine LiquiColor^®^ test, or Urea Nitrogen (BUN) Liqui-UV^®^ (Rate), respectively (EKF Stanbio, United States). The plasma concentration of iFGF23 (Immutopics International, United States) and 1,25(OH)_2_D (Immunodiagnostic Systems Inc, United Kingdom) were measured by enzyme-linked immunosorbent assays according to manufacturers’ protocols.

### RNA Extraction, Reverse Transcription, and qPCR

Organs were harvested and rapidly frozen in liquid nitrogen. Tissues were homogenized using either a BeadBug^TM^ microtube homogenizer or a liquid nitrogen cooled mortar and pestle. Total RNA from bone (one femur and tibia each), bone marrow (spin down from femur and tibia), heart, spleen, and thymus was extracted with TRIzol (Life Technologies Europe B.V., Switzerland) and total RNA from kidney with NucleoSpin^®^ RNA lysis buffer followed by purification with NucleoSpin^®^ RNA Miniprep (Clontech, United States) according to the manufacturers’ protocol including Dnase1 digestion. Total RNA extractions were analyzed for purity and concentration using the NanoDrop ND-1000 spectrophotometer. RNA samples were diluted to a final concentration of 100 ng/μl and cDNA was prepared with reagents from Invitrogen (United States) if not stated otherwise. In brief, in a reaction volume of 40 μl, 300 ng of RNA was used as template and mixed with the following final concentrations of RT buffer (1×): MgCl_2_ (5.5 mmol/l), random hexamers (2.5 μmol/l), dNTP mix (500 μmol/l each; Bioline Ltd., United States), RNase inhibitor (0.4 U/μl), multiscribe reverse transcriptase (1.25 U/μl), and RNAse-free water. Reverse transcription was performed with temperature conditions set at 25°C for 10 min, 48°C for 30 min, and 95°C for 5 min on a thermocycler (Eppendorf). Quantitative PCR (qPCR) was performed using the ABI PRISM 7900HT Detection System (Applied Biosystems). Primers were designed using Primer 3 software (Untergasser et al., 2012). Mouse primer/probe sequences: *Tnf* Fwd 5′-CAGACCCTCACACTCAGATCATCT-3′, Rev 5′-CCTCCACTTGGTGGTTTGCT-3′, Probe 5′-ATTCGAGTGACAAGCCTGTAGCCCACGT-3′; *Tgfb1* Fwd 5′-CCGCAACAAGCCATCTATG-3′, Rev 5′-TGCTTCCCGAATGTCTGACG-3′; *Klotho* Fwd 5′-CAGCTCCAGGCTCGGGTA-3′, Rev 5′-AGGTGTTGTAGAGATGCCAGACTTT-3′, Probe 5′-TTGCCCACAACCTACTTTGGCTCATG-3′; *Vdr* Fwd 5′-CACAAGACCTACGACCCCAC-3′, Rev 5′-CCGGTTCCATCATGTCCAGT-3′; *Gus* Fwd 5′-CTCATCTGGAATTTCGCCGA-3′, Rev 5′-GGCGAGTGAAGATCCCCTTC-3′, Probe 5′-CGAACCAGTCACCGCTGAGAGTAATCG-3′; *Fgf23* Fwd 5′-GACCAGCTATCACCTACAGATCCA-3′, Rev 5′-CGGCGTCCTCTGATGTAATCA-3′, Probe 5′-CCCATCAGACCATCTACAGTGCCCTGA-3′; *Cyp27b1* Fwd 5′-CCTCTGCCGAGACTGGGA-3′, Rev 5′-TCCCGAAAAAGGAAGTGGGT-3′, Probe 5′-TGTTTGCCTTTGCCCAGAGGCAC-3′; *Cyp24a1* Fwd 5′-TACGCTGCTGTCACGGAGC-3′, Rev 5′-TCTGGATTTCCCGGAGAAGTC-3′, Probe 5′-CAGTGGAGACGACCGCAAACAGCTT-3′;. Primers and probes were purchased either from Elim Biopharma (United States), IDT (United States), or Applied Biosystems (Eukaryotic 18S rRNA Endogenous Control primer probe set). Probes were labeled with the reporter dye FAM at the 5′-end and the quencher dye TAMRA or BHQ1 at the 3′-end. qPCR reactions were performed using the TaqMan Fast Advanced Master Mix or PowerUp SYBR Green Master Mix (Applied Biosystems, United States).

### Statistical Analysis

Statistics were performed using unpaired Student‘s *t*-test and linear regression with Pearson correlation (GraphPad Prism version 7, GraphPad, San Diego, CA, United States). *P* < 0.05 was considered significant.

## Results

### iFGF23, Vitamin D and Inflammation in FA-AKI

**FIGURE 1:**
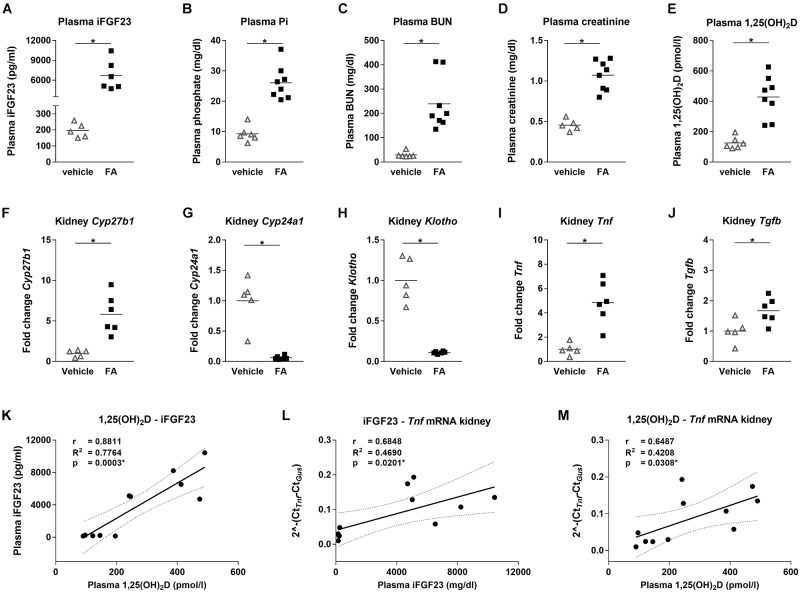
Mineral homeostasis, renal function and inflammation parameters in mice with FA-AKI. **(A)** Plasma iFGF23, **(B)** phosphate, **(C)** BUN, **(D)** creatinine and **(E)** 1,25(OH)_2_D levels, as well as relative renal **(F)** *Cyp27b1*, **(G)** *Cyp24a1*, **(H)** *Klotho*, **(I)** *Tnf* and **(J)** *Tgfb* mRNA expression in vehicle (open triangle) and FA (black squares) treated mice after 24 h. *Gus* was used as housekeeping gene and values were normalized to vehicle group. Five to eight mice per group. Student’s *t*-test ^∗^ *p* < 0.05. Pearson correlation, *R*^2^- and *p*-values of linear regression analysis with 95% confidence interval for **(K)** plasma 1,25(OH)_2_D and iFGF23, **(L)** plasma iFGF23 and renal *Tnf* mRNA expression, **(M)** plasma 1,25(OH)_2_D and renal *Tnf* mRNA expression. Ten to eleven mice. ^∗^*p* < 0.05.

We administered wild type mice with FA or vehicle by intraperitoneal injections to determine the effect of FA-AKI on *Fgf23* gene expression. Twenty-four hours after AKI induction, plasma iFGF23 and phosphate increased significantly while kidney function declined in FA-treated mice (Figures 1A–D). Further, plasma 1,25(OH)_2_D increased by 3-fold in FA-treated mice which was accompanied by 6-fold increase in renal *Cyp27b1* and 17-fold reduction in *Cyp24a1* mRNA expression (Figures 1E–G). FA-AKI reduced *Klotho* mRNA expression and triggered renal inflammation as demonstrated by increased *Tnf* and *Tgfb* mRNA expression (Figures 1H–J). We performed linear regression analyses to determine the relationships between plasma iFGF23, 1,25(OH)_2_D, and inflammation in FA-AKI. Plasma iFGF23 significantly correlated with plasma 1,25(OH)_2_D and both plasma iFGF23 and 1,25(OH)_2_D significantly correlated with renal mRNA expression of the inflammatory cytokine *Tnf* (Figures 1K–M).

### Multiple Organs Contribute to Rapidly Increased iFGF23 in FA-AKI Mice

**FIGURE 2:**
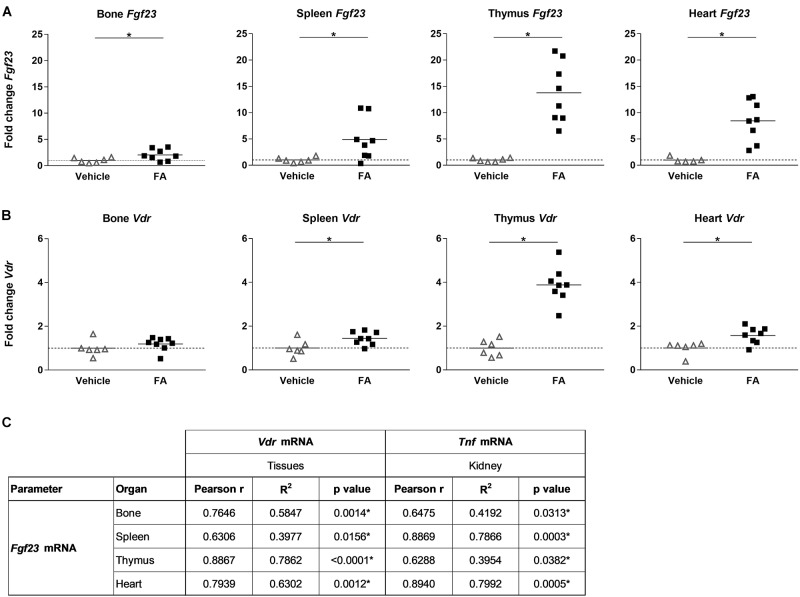
*Fgf23* and *Vdr* mRNA expression in different organs in mice with FA-AKI and correlation between tissue specific *Fgf23*, *Vdr* and renal *Tnf* expression. Relative **(A)** *Fgf23* and **(B)** *Vdr* mRNA expression in bone, spleen, thymus and heart in vehicle (triangle) and FA-treated (squares) mice after 24 h. *18SrRNA* (bone, spleen, thymus) or *Gus* (heart) were used as housekeeping genes and values were normalized to vehicle group. Five to eight mice per group. Student’s *t*-test ^∗^*p* < 0.05. **(C)** Pearson correlation, *R*^2^- and *p*-values of linear regression analysis for *Fgf23* and *Vdr* mRNA expression within the same organ as well as between organ specific *Fgf23* mRNA and renal *Tnf* mRNA expression. Ten to eleven mice. ^∗^*p* < 0.05.

As previously shown, *Fgf23* mRNA expression was upregulated by 2-fold in bone in FA-treated mice compared to the control group (Figure 2A). Furthermore, *Fgf23* mRNA expression increased significantly in spleen, heart and thymus, by 5-, 8-, and 14-fold, respectively. Ectopic renal *Fgf23* mRNA expression was detected in FA-treated mice while it was absent in the control group (not shown). There was no change in *Fgf23* mRNA expression in whole bone marrow with FA treatment (not shown). Previous studies have shown that activation of vitamin D receptor (VDR) by 1,25(OH)_2_D upregulates *Fgf23* expression in bone (Liu et al., 2006). We determined the effect of increased plasma 1,25(OH)_2_D on *Vdr* mRNA expression in the organs we analyzed *Fgf23* mRNA expression. We observed a 1.5- to 4-fold upregulation of *Vdr* mRNA expression in FA-treated mice in spleen, heart and thymus but there was no change in gene expression levels in bone (Figure 2B). *Fgf23* mRNA expression in thymus, spleen, heart, and bone significantly correlated with *Vdr* mRNA expression within the same organ as well as with renal *Tnf* mRNA expression (Figure 2C).

## Discussion

In patients with AKI, higher FGF23 levels are associated with AKI progression and increased mortality but mechanisms by which circulating FGF23 increase with kidney injury are unknown (Leaf et al., 2013, 2016, 2017). In the FA-AKI mouse model, increased *Fgf23* mRNA in bone was shown to contribute to the increase in plasma FGF23 (Christov et al., 2013). In this study, we demonstrate that increased *Fgf23* mRNA expression in multiple organs including bone accompanies the rapid increase in plasma iFGF23 in FA-AKI mice. Furthermore the rise in plasma iFGF23 is paralleled by increased plasma 1,25(OH)_2_D. Interestingly, the two lymphoid organs, thymus and spleen, show a very high induction of *Fgf23* mRNA expression in mice with FA-AKI. The pattern of *Fgf23* expression in lymphoid organs and its rapid increase following AKI suggest that FGF23 plays an important role in the inflammatory response triggered by FA-AKI including increased *Tnf* expression and elevated plasma IL-6 (Moreno et al., 2011; Wen et al., 2012). We confirmed that FA-AKI increases renal mRNA expression of inflammatory cytokines, *Tnf* and *Tgfb*. Moreover, we demonstrate that plasma iFGF23, 1,25(OH)_2_D and *Fgf23* mRNA expression significantly correlated with renal *Tnf* mRNA expression. Inflammatory stimuli such as LPS, IL-1β, and TNF have been shown to increase *Fgf23* mRNA expression *in vivo* and *in vitro* (Ito et al., 2015; Masuda et al., 2015; David et al., 2016). In mice with LPS-induced intermittent chronic inflammation, *Fgf23* mRNA expression in spleen is increased and splenectomy reduces plasma FGF23 levels (Bansal et al., 2017). Whether these cytokines upregulate *Fgf23* mRNA expression in spleen and thymus in FA-AKI need further evaluation.

Ectopic renal FGF23 expression has been reported in animal models of chronic kidney disease and in mice with unilateral ureter obstruction (Zanchi et al., 2013; Spichtig et al., 2014; Smith et al., 2017). Renal FGF23 expression in uremic rats does not contribute to increased circulatory FGF23 levels but local production may further trigger renal fibrosis in these animals (Mace et al., 2017; Smith et al., 2017). Our findings of renal *Fgf23* mRNA expression in FA-treated mice expands the pathological conditions where ectopic renal FGF23 expression is present. Hemorrhagic shock and sepsis-induced AKI in rats result in EPO-EPO receptor-dependent upregulation of *Fgf23* mRNA levels in the bone marrow (Toro et al., 2018). In our study, we did not detect any changes in *Fgf23* mRNA expression in whole bone marrow of FA-AKI mice. AKI disrupts the crosstalk between kidney and heart and contributes to acute reno-cardiac syndrome (Kingma et al., 2015). The resulting cardiac dysfunction is attributed to changes in kidney function, hemodynamics and inflammatory mediators (Kingma et al., 2015). Further studies are needed to determine whether the inflammatory response caused by FA-AKI is responsible for the increase in *Fgf23* mRNA expression in the heart of FA-AKI mice.

In bone, 1,25(OH)_2_D activates VDR and allows VDR binding to VDR response elements in the *Fgf23* promoter region to upregulate *Fgf23* mRNA expression (Liu et al., 2006). Therefore, increase in local VDR expression together with high plasma 1,25(OH)_2_D may be responsible for increased *Fgf23* mRNA expression seen in different organs in FA-treated mice. Indeed, we found elevated plasma 1,25(OH)_2_D in FA-AKI mice and increased *Vdr* mRNA expression in spleen, thymus and heart but not in bone. Moreover, *Vdr* mRNA expression strongly correlated with *Fgf23* mRNA expression in the same organ. Therefore, activation of 1,25(OH)_2_D-VDR signaling pathway may contribute to increase in plasma iFGF23 in FA-AKI mice.

Increased plasma 1,25(OH)_2_D in FA-AKI mice was accompanied by increased renal *Cyp27b1* and decreased *Cyp24a1* mRNA expression. These results suggest that FGF23 is unable to regulate renal vitamin D metabolism in FA-AKI mice which could be partially explained by interrupted FGF23 signaling due to a decrease in renal Klotho expression with AKI (Hu et al., 2010). Similar to our study, 3/4 nephrectomized rats also demonstrate increased renal *Cyp27b1* and decreased *Cyp24a1* and *Vdr* mRNA expression (Takemoto et al., 2003). However, in patients with CKD and acute renal inflammation plasma 1,25(OH)_2_D is low with increased *CYP27b1*, *CYP24a1*, and *VDR* expression (Zehnder et al., 2008). Renal *CYP27b1* expression in these patients was localized to activated macrophages and epithelial cells (Zehnder et al., 2008). Therefore, humans and rodents differ in the renal expression patterns for genes in the vitamin D metabolic pathway.

In summary, we demonstrate that the rapid rise in plasma iFGF23 in mice with FA-AKI is accompanied by increased *Fgf23* mRNA expression in multiple organs including bone, thymus, spleen, heart and kidney. Furthermore, increased plasma iFGF23 levels are paralleled by increased plasma 1,25(OH)_2_D and increased *Vdr* mRNA expression in thymus, spleen, and heart.

## Author Contributions

DE-S and FP conceived the study, provided the methodology, and wrote, reviewed, and edited the manuscript. DE-S performed the formal analysis and visualization process and wrote the original draft of the manuscript. DE-S and MZ contributed to the investigation process. FP gathered resources and supervised the study and acquired funding. All authors read, edited, and approved the manuscript.

## Conflict of Interest Statement

The authors declare that the research was conducted in the absence of any commercial or financial relationships that could be construed as a potential conflict of interest.

## References

[B1] Bansal S. Friedrichs W. E. Velagapudi C. Feliers D. Khazim K. Horn D. (2017). Spleen contributes significantly to increased circulating levels of fibroblast growth factor 23 in response to lipopolysaccharide-induced inflammation. *Nephrol. Dial. Transplant.* 32 960–968. 10.1093/ndt/gfw376 27836924 PMC6075072

[B2] Chen G. Liu Y. Goetz R. Fu L. Jayaraman S. Hu M. C. (2018). alpha-Klotho is a non-enzymatic molecular scaffold for FGF23 hormone signalling. *Nature* 553 461–466. 10.1038/nature25451 29342138 PMC6007875

[B3] Christov M. Waikar S. S. Pereira R. C. Havasi A. Leaf D. E. Goltzman D. (2013). Plasma FGF23 levels increase rapidly after acute kidney injury. *Kidney Int.* 84 776–785. 10.1038/ki.2013.150 23657144 PMC3766419

[B4] David V. Martin A. Isakova T. Spaulding C. Qi L. Ramirez V. (2016). Inflammation and functional iron deficiency regulate fibroblast growth factor 23 production. *Kidney Int.* 9 135–146. 10.1038/ki.2015.290 26535997 PMC4854810

[B5] Fon Tacer K. Bookout A. L. Ding X. Kurosu H. John G. B. Wang L. (2010). Research resource: comprehensive expression atlas of the fibroblast growth factor system in adult mouse. *Mol. Endocrinol.* 24 2050–2064. 10.1210/me.2010-0142 20667984 PMC2954642

[B6] Hanudel M. R. Wesseling-Perry K. Gales B. Ramos G. Campbell V. Ethridge K. (2016). Effects of acute kidney injury and chronic hypoxemia on fibroblast growth factor 23 levels in pediatric cardiac surgery patients. *Pediatr. Nephrol.* 31 661–669. 10.1007/s00467-015-3257-5 26525200 PMC4766020

[B7] Hassan A. Durlacher K. Silver J. Naveh-Many T. Levi R. (2016). The fibroblast growth factor receptor mediates the increased FGF23 expression in acute and chronic uremia. *Am. J. Physiol. Renal Physiol.* 310 F217–F221. 10.1152/ajprenal.00332.2015 26311115

[B8] Hu M. C. Shi M. Zhang J. Quiñones H. Kuro-o M. Moe O. W. (2010). Klotho deficiency is an early biomarker of renal ischemia-reperfusion injury and its replacement is protective. *Kidney Int.* 78 1240–1251. 10.1038/ki.2010.328 20861825 PMC3237296

[B9] Ito N. Wijenayaka A. R. Prideaux M. Kogawa M. Ormsby R. T. Evdokiou A. (2015). Regulation of FGF23 expression in IDG-SW3 osteocytes and human bone by pro-inflammatory stimuli. *Mol. Cell. Endocrinol.* 399 208–218. 10.1016/j.mce.2014.10.007 25458698

[B10] Kingma J. G. Simard D. Rouleau J. R. (2015). Renocardiac syndromes: physiopathology and treatment stratagems. *Can. J. Kidney Health Dis.* 2:41. 10.1186/s40697-015-0075-4 26478820 PMC4608312

[B11] Kurosu H. Ogawa Y. Miyoshi M. Yamamoto M. Nandi A. Rosenblatt K. P. (2006). Regulation of fibroblast growth factor-23 signaling by klotho. *J. Biol. Chem.* 281 6120–6123. 10.1074/jbc.C500457200 16436388 PMC2637204

[B12] Leaf D. E. Christov M. Jüppner H. Siew E. Ikizler T. A. Bian A. (2016). Fibroblast growth factor 23 levels are elevated and associated with severe acute kidney injury and death following cardiac surgery. *Kidney Int.* 89 939–948. 10.1016/j.kint.2015.12.035 26924052 PMC4801748

[B13] Leaf D. E. Jacob K. A. Srivastava A. Chen M. E. Christov M. Jüppner H. (2017). Fibroblast growth factor 23 levels associate with AKI and death in critical illness. *J. Am. Soc. Nephrol.* 28 1877–1885. 10.1681/ASN.2016080836 28028134 PMC5461795

[B14] Leaf D. E. Waikar S. S. Wolf M. Cremers S. Bhan I. Stern L. (2013). Dysregulated mineral metabolism in patients with acute kidney injury and risk of adverse outcomes. *Clin. Endocrinol.* 79 491–498. 10.1111/cen.12172 23414198 PMC3686895

[B15] Liu S. Tang W. Zhou J. Stubbs J. R. Luo Q. Pi M. (2006). Fibroblast growth factor 23 is a counter-regulatory phosphaturic hormone for vitamin D. *J. Am. Soc. Nephrol.* 17 1305–1315. 10.1681/ASN.2005111185 16597685

[B16] Mace M. L. Gravesen E. Nordholm A. Hofman-Bang J. Secher T. Olgaard K. (2017). Kidney fibroblast growth factor 23 does not contribute to elevation of its circulating levels in uremia. *Kidney Int.* 92 165–178. 10.1016/j.kint.2017.01.015 28341272

[B17] Masuda Y. Ohta H. Morita Y. Nakayama Y. Miyake A. Itoh N. (2015). Expression of fgf23 in activated dendritic cells and macrophages in response to immunological stimuli in mice. *Biol. Pharm. Bull.* 38 687–693. 10.1248/bpb.b14-00276 25739891

[B18] Moreno J. A. Izquierdo M. C. Sanchez-Niño M. D. Suárez-Alvarez B. Lopez-Larrea C. Jakubowski A. (2011). The inflammatory cytokines TWEAK and TNFa reduce renal klotho expression through NFkB. *J. Am. Soc. Nephrol.* 22 1315–1325. 10.1681/ASN.2010101073 21719790 PMC3137579

[B19] Ohyama Y. Noshiro M. Okuda K. (1991). Cloning and expression of cDNA encoding 25-hydroxyvitamin D3 24-hydroxylase. *FEBS Lett.* 278 195–198. 10.1016/0014-5793(91)80115-J 1991512

[B20] Perwad F. Zhang M. Y. Tenenhouse H. S. Portale A. A. (2007). Fibroblast growth factor 23 impairs phosphorus and vitamin D metabolism in vivo and suppresses 25-hydroxyvitamin D-1α-hydroxylase expression in vitro. *Am. J. Physiol. Renal. Physiol.* 293 F1577–F1583. 10.1152/ajprenal.00463.2006 17699549

[B21] Ranch D. Zhang M. Y. Portale A. A. Perwad F. (2011). Fibroblast growth factor 23 regulates renal 1,25-dihydroxyvitamin D and phosphate metabolism via the MAP kinase signaling pathway in Hyp mice. *J. Bone Miner. Res.* 26 1883–1890. 10.1002/jbmr.401 21472778 PMC4409871

[B22] Shimada T. Hasegawa H. Yamazaki Y. Muto T. Hino R. Takeuchi Y. (2004). FGF-23 is a potent regulator of vitamin D metabolism and phosphate homeostasis. *J. Bone Miner. Res.* 19 429–435. 10.1359/JBMR.0301264 15040831

[B23] Smith E. R. Tan S. J. Holt S. G. Hewitson T. D. (2017). FGF23 is synthesised locally by renal tubules and activates injury-primed fibroblasts. *Sci. Rep.* 7:3345. 10.1038/s41598-017-02709-w 28611350 PMC5469734

[B24] Speer T. Groesdonk H. V. Zapf B. Buescher V. Beyse M. Duerr L. (2015). A single preoperative FGF23 measurement is a strong predictor of outcome in patients undergoing elective cardiac surgery: a prospective observational study. *Crit. Care* 19:190. 10.1186/s13054-015-0925-6 25902817 PMC4424828

[B25] Spichtig D. Zhang H. Mohebbi N. Pavik I. Petzold K. Stange G. (2014). Renal expression of FGF23 and peripheral resistance to elevated FGF23 in rodent models of polycystic kidney disease. *Kidney Int.* 85 1340–1350. 10.1038/ki.2013.526 24402093

[B26] Takemoto F. Shinki T. Yokoyama K. Inokami T. Hara S. Yamada A. (2003). Gene expression of vitamin D hydroxylase and megalin in the remnant kidney of nephrectomized rats. *Kidney Int.* 64 414–420. 10.1046/j.1523-1755.2003.00114.x 12846736

[B27] Takeyama K. Kitanaka S. Sato T. Kobori M. Yanagisawa J. Kato S. (1997). 25-hydroxyvitamin D3 1α-hydroxylase and vitamin D synthesis. *Science* 277 1827–1830. 10.1126/science.277.5333.1827 9295274

[B28] Toro L. Barrientos V. León P. Rojas M. Gonzalez M. González-Ibáñez A. (2018). Erythropoietin induces bone marrow and plasma fibroblast growth factor 23 during acute kidney injury. *Kidney Int.* 93 1131–1141. 10.1016/j.kint.2017.11.018 29395333

[B29] Untergasser A. Cutcutache I. Koressaar T. Ye J. Faircloth B. C. Remm M. (2012). Primer3–new capabilities and interfaces. *Nucleic Acids Res.* 40 e115. 10.1093/nar/gks596 22730293 PMC3424584

[B30] Wen X. Peng Z. Li Y. Wang H. Bishop J. V. Chedwick L. R. (2012). One dose of cyclosporine A is protective at initiation of folic acid-induced acute kidney injury in mice. *Nephrol. Dial. Transplant.* 27 3100–3109. 10.1093/ndt/gfr766 22294776 PMC3408935

[B31] Zanchi C. Locatelli M. Benigni A. Corna D. Tomasoni S. Rottoli D. (2013). Renal expression of FGF23 in progressive renal disease of diabetes and the effect of ace inhibitor. *PLoS One* 8:e70775. 10.1371/journal.pone.0070775 23967103 PMC3743899

[B32] Zehnder D. Quinkler M. Eardley K. S. Bland R. Lepenies J. Hughes S. V. (2008). Reduction of the vitamin D hormonal system in kidney disease is associated with increased renal inflammation. *Kidney Int.* 74 1343–1353. 10.1038/ki.2008.453 18784644 PMC2737358

